# HealthTrust: A Social Network Approach for Retrieving Online Health Videos

**DOI:** 10.2196/jmir.1985

**Published:** 2012-01-31

**Authors:** Luis Fernandez-Luque, Randi Karlsen, Genevieve B Melton

**Affiliations:** ^1^Northern Research InstituteTromsøNorway; ^2^Computer Science DepartmentUniversity of TromsøTromsøNorway; ^3^Institute for Health InformaticsUniversity of MinnesotaMinneapolis, MNUnited States

**Keywords:** Medical informatics, information storage and retrieval, video, online systems, health communication, diabetes

## Abstract

**Background:**

Social media are becoming mainstream in the health domain. Despite the large volume of accurate and trustworthy health information available on social media platforms, finding good-quality health information can be difficult. Misleading health information can often be popular (eg, antivaccination videos) and therefore highly rated by general search engines. We believe that community wisdom about the quality of health information can be harnessed to help create tools for retrieving good-quality social media content.

**Objectives:**

To explore approaches for extracting metrics about authoritativeness in online health communities and how these metrics positively correlate with the quality of the content.

**Methods:**

We designed a metric, called HealthTrust, that estimates the trustworthiness of social media content (eg, blog posts or videos) in a health community. The HealthTrust metric calculates reputation in an online health community based on link analysis. We used the metric to retrieve YouTube videos and channels about diabetes. In two different experiments, health consumers provided 427 ratings of 17 videos and professionals gave 162 ratings of 23 videos. In addition, two professionals reviewed 30 diabetes channels.

**Results:**

HealthTrust may be used for retrieving online videos on diabetes, since it performed better than YouTube Search in most cases. Overall, of 20 potential channels, HealthTrust’s filtering allowed only 3 bad channels (15%) versus 8 (40%) on the YouTube list. Misleading and graphic videos (eg, featuring amputations) were more commonly found by YouTube Search than by searches based on HealthTrust. However, some videos from trusted sources had low HealthTrust scores, mostly from general health content providers, and therefore not highly connected in the diabetes community. When comparing video ratings from our reviewers, we found that HealthTrust achieved a positive and statistically significant correlation with professionals (Pearson *r*_10_ = .65, *P* = .02) and a trend toward significance with health consumers (*r*_7_ = .65, *P* = .06) with videos on hemoglobinA_1__c_, but it did not perform as well with diabetic foot videos.

**Conclusions:**

The trust-based metric HealthTrust showed promising results when used to retrieve diabetes content from YouTube. Our research indicates that social network analysis may be used to identify trustworthy social media in health communities.

## Introduction

The Internet is emerging as one of the main sources of consumer health information [1,2]. Many health authorities, medical associations, hospitals, and patients have published or are publishing online content, including through social media platforms (eg, blogs, YouTube, or Twitter). Kaplan and Haenlein defined social media as consisting of a “set of Web applications, which allows the creation and exchange of user-generated content” [3]. Social media are becoming increasingly mainstream in the health domain [4-6]. For example, there are more than 500 channels on YouTube created by American hospitals, containing thousands of videos [7]. Similarly, the United Kingdom’s National Health Service has published more than 500 videos on YouTube [8].

Despite the large volume of good-quality health information available on social media platforms, finding accurate and trustworthy health information can be surprisingly difficult [9-13]. There is a great deal of misinformation, and one often comes across content promoting anorexia or avoiding vaccinations [14,15]. Sometimes bogus health information can become extremely popular and viral (eg, conspiracy theories about vaccination). Therefore, sifting through this to find trustworthy health information remains one of the main challenges faced by health consumers.

In conjunction with the large quantity of information, many health consumers rely on online communities for relevant information. Indeed, online health communities have been found to be very effective in filtering misleading information [16]. Members of online communities have to build their trust gradually, which makes it hard for sources that are not trusted to disseminate misinformation. It is also possible to ask peers about high-quality health information; however, peers are not available all the time and often cannot provide instant feedback.

The objective of this study was to explore approaches for extracting metrics about authoritativeness in online health communities and how these metrics would positively correlate with the quality of the content. An authoritative member of the community (such as the American Diabetes Association) tends to publish or endorse content of better quality than do nonauthoritative members of the community. Using link-based analysis, we extracted a metric (called HealthTrust) about authoritativeness in a health community. We then implemented an algorithm for searching videos and channels based on HealthTrust and tested it with online diabetes content from YouTube.

### Background

Outside of the health domain, human experts are rarely used in any scalable fashion for classifying and retrieving webpages. Web information retrieval systems rely on automatic approaches to harvest reputable online resources, mainly based on the analysis of links between pages [17-21]. In Google’s PageRank, links from one site to another can be modeled as an endorsement, and they are used to calculate a global rank of all the websites [18]. Another example is the hyperlink-induced topic search (HITS) algorithm [17]. As explained in the following section, HITS is a link analysis algorithm for ranking webpages based on two scores: authoritativeness and hubs. Hubs are essentially webpages that function as directories that have links to authoritative pages. The authorities are webpages that are linked by many of the most representative Webs, so they have a high authoritativeness within the community of Webs. Other algorithms, such as TrustRank, take into account trustworthiness in online communities, aimed at making the search more robust to Web spam [20]. Gou et al explored how to use social network analysis for ranking online videos in a personalized manner [21]. Mislove et al studied the integration of general-purpose social networks with online Web searches [22].

To our knowledge none of those algorithms have been studied in the health domain. One of the main challenges in the health domain is that misleading health information can be very popular (eg, antivaccination videos) and therefore may be paradoxically highly rated and not considered spam by general information retrieval algorithms.

Health consumers need tailored tools to help them find good-quality health social media and websites. A common approach consists in creating quality labels for trustworthy health websites that adhere to a set of guidelines [8,23,24]. Some studies have pointed out cases where those guidelines were not that effective for finding good health information [10,25]. Another difficulty is choosing among dozens of guidelines [23,24]. These guidelines have been combined with automatic approaches that extract certain quality indicators [11,12,26-28] used for online health information retrieval. However, automatic approaches are still not widely used. To our knowledge, none of these projects focus on link-based analysis and trust metrics of health websites, as generic search engines do. In addition, despite the popularity of health videos, we have not come across any project specifically aimed at developing tools to help find relevant health videos.

## Methods

In the next subsection, we describe the metric HealthTrust and how it can be integrated to enhance the search of social media content (ie, YouTube diabetes videos). In the subsequent subsections, we describe two studies aimed at evaluating the relationship between the HealthTrust scores of diabetes videos and channels, and their quality as perceived by end users. We designed these experiments to evaluate our hypothesis that HealthTrust’s metric can be used to improve the retrieval of health social media. In the first study, we evaluated the use of HealthTrust for filtering diabetes channels from YouTube. In a second study, we evaluated the correlation between HealthTrust scores and ratings of videos about diabetes *A* *1* *c test* *ing* and *diabetic foot*.

### HealthTrust

According to the Merriam Webster Dictionary, trust is an “assured reliance on the character, ability, strength, or truth of someone or something” [29]. Other related terms, treated as equivalent to *trust*, are *authoritativeness* (“clearly accurate or knowledgeable” [30]) and *reputation* (“overall quality or character as seen or judged by people in general” [31]). In the Web information retrieval domain, trust has normally been based on the analysis of link structures. A link from one website to another implies an endorsement of the linked website; this approach is very similar to the calculation of impact factors for journals. Trust in the health domain is mainly related to the concept of authoritativeness in terms of the reliability and knowledge of the content creator. There are, however, many additional aspects related to trust such as *appearance* and *impartiality* [32]. Within the scope of this study, we define trust as the “assured reliance on the quality of users and content within an online health community.”

As we mentioned in the introduction, online health communities can be effective in filtering out misleading health information [16]. Users disseminating misleading information have a hard job gaining trust within the community. A user creating videos about herbal cures for diabetes will receive less endorsement from the diabetes community than a video created by the American Diabetes Association.

We assume that misleading information will be less endorsed within the health community. Consequently, trustworthiness within the health community will correlate with higher content quality. To compute the trustworthiness of health social media, we designed an algorithm to calculate a metric, called HealthTrust, that estimates the trustworthiness of social media content (eg, blog post or video) in the health community to which it belongs. To evaluate HealthTrust we designed an algorithm for searching online health videos based on that metric.

#### HealthTrust Metric

**Figure 1:**
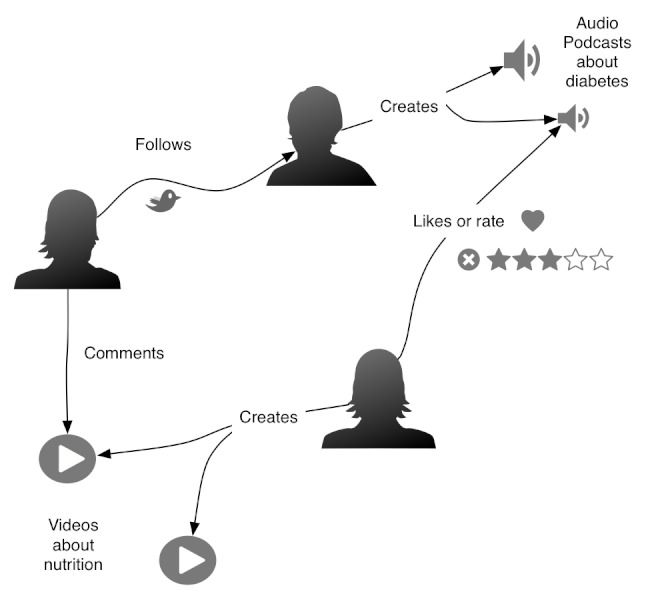
Example of a health social network.

Users and content are heavily interconnected in the context of health social media. Figure 1 shows that links between users and content form a graph that models a social network where it is possible to calculate trust-related metrics. Content and users are interconnected and can form a health community with a common interest (eg, diabetes).

**Figure 2:**
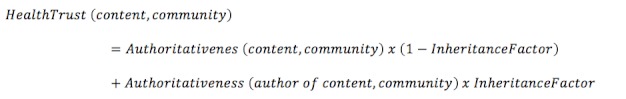
Calculation of the HealthTrust content score.

HealthTrust (Figure 2) is a metric about trust of content and members of a health community. Trust can be modeled for both users (“I trust this author”) and content (“I trust this content”). In fact, your trust in a particular piece of content should be a combination of how much you trust its author and the content itself. Based on these considerations we designed the HealthTrust metric. To calculate HealthTrust a set of steps must be followed: (1) extraction of the community where HealthTrust is going to be applied, (2) calculation of the authoritativeness scores for content and users based on their links, and (3) calculation of HealthTrust scores. Finally, this score can be used for information retrieval purposes as explained in the subsection “HealthTrust for Search.”

##### Community Extraction

HealthTrust is applied to only a certain health community. That community can be identified by many different means, such as manual selection of users and heuristic approaches [33]. As explained in the following section, in our study we extracted YouTube users interested in diabetes by using different search queries related to diabetes. Community extraction is a core aspect in HealthTrust, since the metric is not calculating the general authoritativeness of the content but rather the authoritativeness in a particular community. In the case of YouTube in general, MTV videos from rock stars may be more authoritative than videos from health agencies such as the US Centers for Disease Control and Prevention (CDC). On the contrary, with HealthTrust the focus is on intracommunity authoritativeness. For example, in the health community the CDC is far more authoritative than MTV.

**Figure 3:**
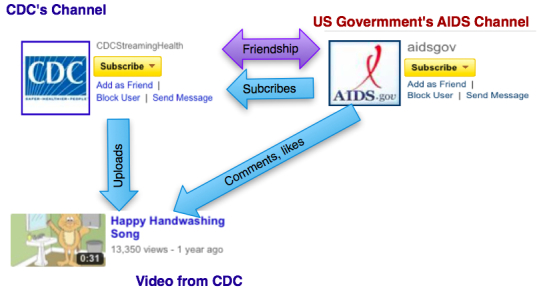
YouTube’s social network. CDC = Centers for Disease Control and Prevention.

For our case study we used the diabetes community on the online video-sharing platform YouTube. As shown on Figure 3, YouTube can be modeled as a social network where users (ie, channels) can build their reputation using different social links (eg, subscriptions, friendships, favorite videos, or comments) [34]. In our study, we took into consideration favorite videos and subscriptions, since these links are the most commonly used by all members of the community.

In our first study we used the YouTube application programming interface (API) to search all the channels that had the keyword *diabetes* and extracted all the accessible information about them (eg, uploads, subscriptions, and favorites). In our second study, we extracted community searches for videos using a set of diabetes-related queries. We extracted all the information about these videos and their authors.

##### Authoritativeness Scores

The authoritativeness scores in HealthTrust can be calculated using link-based metrics such as PageRank scores [18] or HITS authoritativeness [17]. As explained in the next section, in our study we used the HITS authoritativeness score. In these algorithms, the links between websites are used to model a bidirectional graph with incoming and outgoing links. A recursive algorithm is used to score the reputation of a website based on the incoming links, since an incoming link is considered an endorsement of the linked website. The HITS algorithm considers two types of nodes: authorities and hubs. The hubs are the nodes that tend to link to the most authoritative webpages. The authoritative scores in HITS are calculated based on the incoming links from hubs.

**Figure 4:**
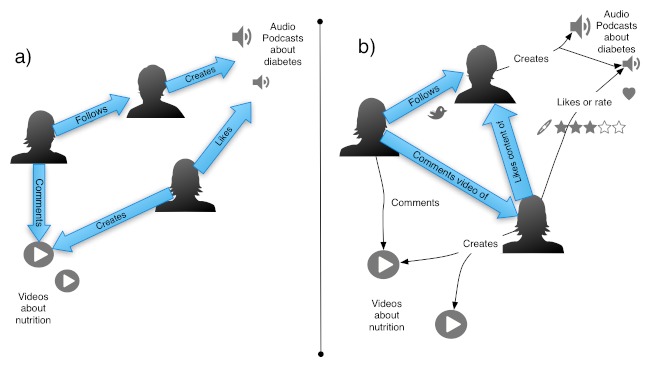
Links (in blue) used to calculate authoritativeness of users (left) and content (right). Diagram based on Figure 1.

The authoritativeness of content and users are calculated as follows. First, the authoritativeness of content (Figure 4, left) is calculated based on the links between all users and content. Both content and users are considered nodes. Second, the authoritativeness of users (Figure 4, right) is calculated based on the links between all users, which are the only nodes. If a user likes or favors content from another user, this is considered as a link between the users.

In our study we used the Java Universal Network/Graph (JUNG) API [35] to calculate the HITS authoritativeness values of users (ie, channels) and content (videos) as follows. First, for the authoritativeness of users, we created a graph where the nodes were the channels and the edges were their subscriptions (channel X subscribed to channel Y) and favorites (channel X subscribed to video of channel Y). Then, that graph was used to calculate the HITS authoritativeness values of the channels. Second, for the authoritativeness scores of videos, we considered videos and channels to be nodes and the edges to be favorites (channel X subscribed to video Z) and subscriptions (channel X subscribed to video of channel Y). That graph was used to calculate the HITS authoritativeness values of the videos.

The authoritativeness values for content and users are calculated independently as shown in the Figure 4. Therefore, to combine them it is necessary to normalize their ranges—for example, in our study we normalized the authoritativeness scores of videos and users between 0 and 1.

##### Calculation of HealthTrust

The HealthTrust score of a particular piece of content (eg, video or blog post) is the weighted combination of the normalized authoritativeness scores of content. The weighted combination is based on the InheritanceFactor. The weighted approach is designed to allow part of the trustworthiness to be inherited by the content from its author. Thus, new content from a trusted author will have a higher HealthTrust score than new content from an untrustworthy author. To give a high weight to the InheritanceFactor implies that the authoritativeness of the author is very important. For example, a video from the CDC will have implicit authoritativeness even if it is new and has never been rated or linked. An InheritanceFactor of 0 implies that there is no inheritance transfer of trust from the authors to their content, so all the authoritativeness is based on the video’s score.

**Figure 5:**
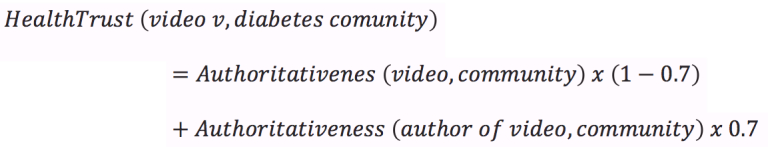
HealthTrust calculation for diabetes videos from YouTube.

As Figure 5 shows, in the video study authoritativeness scores were combined with an InheritanceFactor of 0.7, meaning that the HITS authoritative value of videos weighed 30% and the author’s authoritativeness 70%. We decided on these values after testing with several queries (not used in our evaluation) in a previous data set. We observed that there were many new high-quality videos without links to them, so a lower value for the InheritanceFactor would have decreased their HealthTrust despite being from a trusted content provider.

#### HealthTrust for Search

We believe that HealthTrust can be used to enhance the retrieval of content within health communities. To evaluate that possibility we designed a search algorithm that combines query matching with HealthTrust. Our search algorithm is based on combining two scores: (1) relevance of the content to the search query, and (2) HealthTrust. Relevance can be calculated using simple query matching (eg, the content contains the query in its title or in the description).

**Figure 6:**
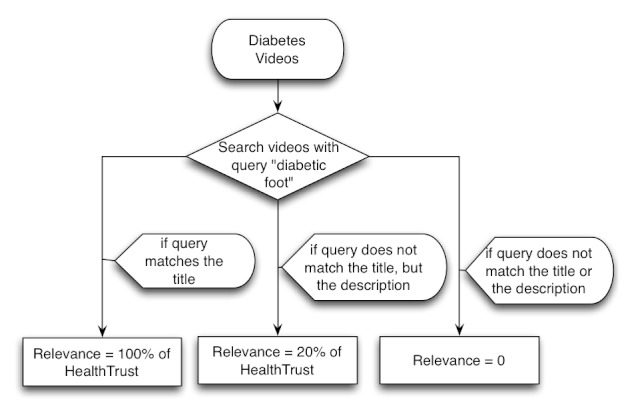
Relevance calculation for HealthTrust-based search.

We implemented a search algorithm based on HealthTrust to study whether that metric may be use to retrieve diabetes videos. As shown in Figure 6, our search algorithm combined the syntactic query match with the HealthTrust values. If the query matched the video’s title the relevance was computed as 100% of the video’s HealthTrust score. If the query only matched the description, the relevance was computed as 20% of the video’s HealthTrust score. We decided on these values after observing the quality of video metadata. In particular, we observed that titles are very important to infer the relevance of videos, since descriptions and tags tend to be very heterogeneous (eg, due to tag spamming). In a previous study, we also found that the quality of comments on YouTube health videos can be very heterogeneous [36].

### Study: Diabetes Channels and HealthTrust

As described in a previous report [37], in May 2010, we performed a study to evaluate the feasibility of using social network analysis to filter YouTube diabetes channels. The objective of this study was to test whether the authoritativeness values of the diabetes channels in YouTube are related to their quality.

#### Data Collection

**Figure 7:**
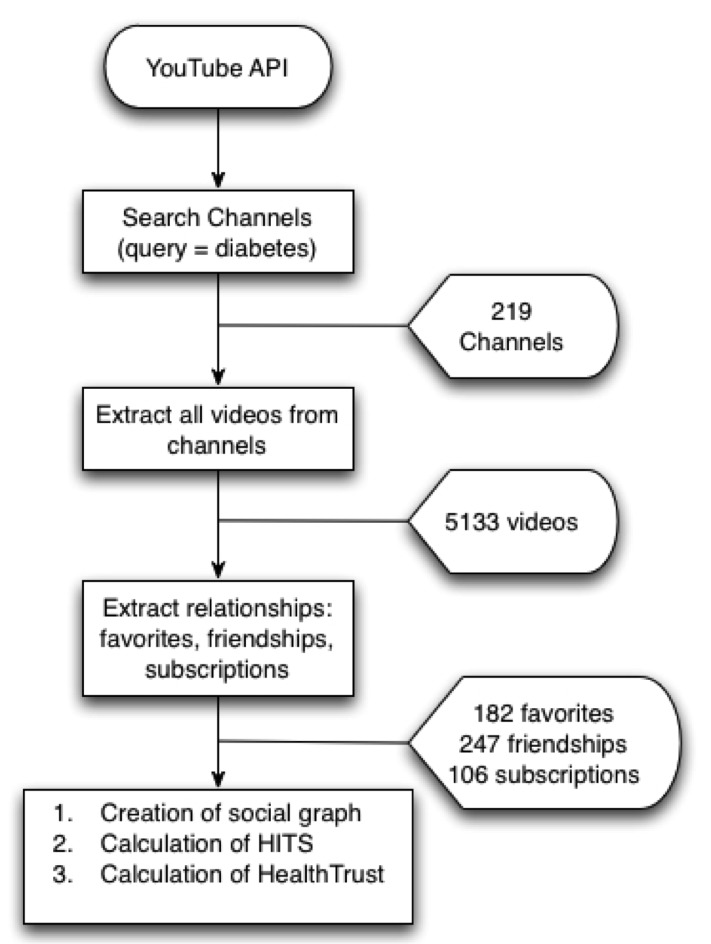
Data extraction in the study of diabetes channels and HealthTrust. API = application programming interface; HITS = hyperlink-induced topic search.

Figure 7 describes how we extracted 5133 videos, 219 channels, 182 favorites, and 247 friendships about diabetes from YouTube using the YouTube API. We searched channels with the query *diabetes* and extracted their information (links, videos, descriptions, etc) to calculate their HealthTrust scores, which corresponded to the authoritativeness values of the channels, since we did not take videos into consideration in this study.

#### Recruitment and Ratings

Two health care professionals rated channels from a list containing the top 20 diabetes channels retrieved by YouTube and HealthTrust’s top 20 channels. The reviewers received a list with all the channels alphabetically ordered and were asked to respond with “yes” or “no” to whether they would recommend the diabetes channel to a patient with diabetes.

The interrated agreement score based on Cohen kappa [38] was calculated using the statistical framework R [39] and resulted in good agreement (.61).

#### Data Analysis

We evaluated the results using the precision at K metric [40], with K being the top-ranked retrieved results. This technique is used widely to evaluate Web search engines, since users tend to use only the top search results. We also evaluated the results with the metric discounted cumulative gain (DCG). DCG is commonly used to evaluate ranked lists of Web search results taking into account the position of the retrieved results [41]. The relevance *gain* decreases logarithmically based on the position of the retrieved results.

### Search Study: Diabetes Videos and HealthTrust

#### Data Collection

**Figure 8:**
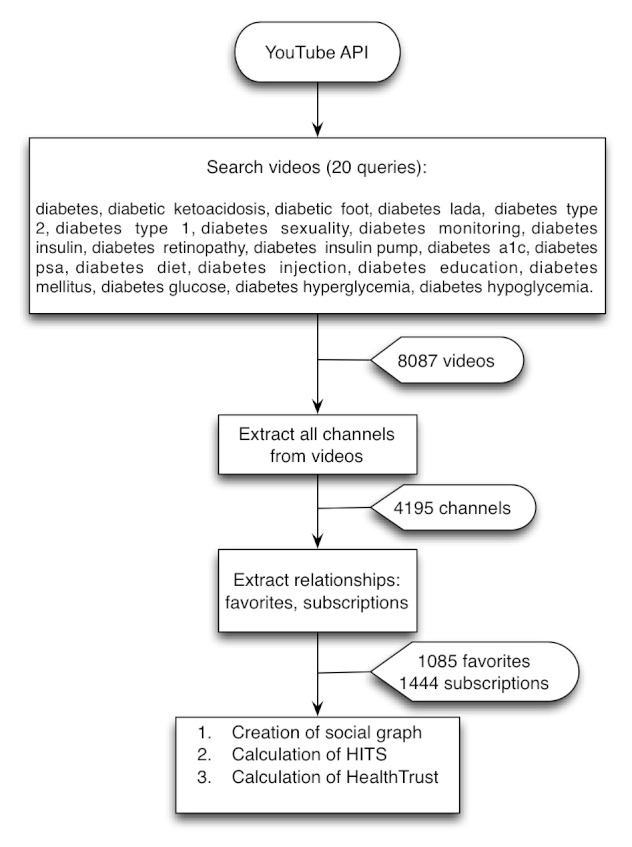
Data extraction on the search study on diabetes videos and HealthTrust. API = application programming interface; HITS = hyperlink-induced topic search.

In April 2011, we collected from YouTube 8087 diabetes videos using the search API with 20 different queries (*diabetic foot*, *diabetes*, *diabetes ketoacidosis*, etc) as explained in Figure 8. We also extracted all the available information about channels, subscriptions, and favorites. Finally, we calculated the HealthTrust scores for videos and channels.

Although our dataset contained videos found by different queries, we evaluated videos from only two queries in order to increase the number of responses per video. We limited our study to the evaluation of searches about two information needs that are important for most people affected by diabetes: diabetes foot issues and hemoglobin A_1__c_ (glycated hemoglobin) testing. Diabetes foot problems are very common among people with diabetes and require a lot of attention to avoid very serious complications that can lead to amputation. Diabetes hemoglobin A_1c_ testing is a very common laboratory test to evaluate how well the diabetes is managed.

Most of the responders rated different videos, since there were four different lists and some of the surveys were not completely filled out. Therefore, there was not enough data to calculate a meaningful interannotator agreement score in this study. For each type of responder (professionals and consumers), we aggregated the ratings of the different videos and calculated the average rating values.

#### Recruitment

After extracting the dataset of diabetes videos we recruited professionals and health consumers to evaluate the results. The recruitment took place between April 25 and June 14, 2011.

We recruited health care professional reviewers using a snowball approach, where invitations were sent to professional mailing lists. We collected 82 informed consents, and 27 video surveys were completed (2 surveys were removed due to the lack of information about the profession of the respondents). In total, professionals provided 162 ratings of 23 videos.

We recruited health consumers from the online diabetes community TuDiabetes.org, which has more than 20,000 members. Information about the study was posted on the community’s main blog and in their mailing list (about 10,000 subscribers). We received 178 informed consents, and 73 surveys were partially or completely filled in. In total, consumers provided 427 ratings of 17 videos. A donation of US $5 per survey was given to the Diabetes Hands Foundation, which runs the online community.

#### Video Surveys

**Figure 9:**
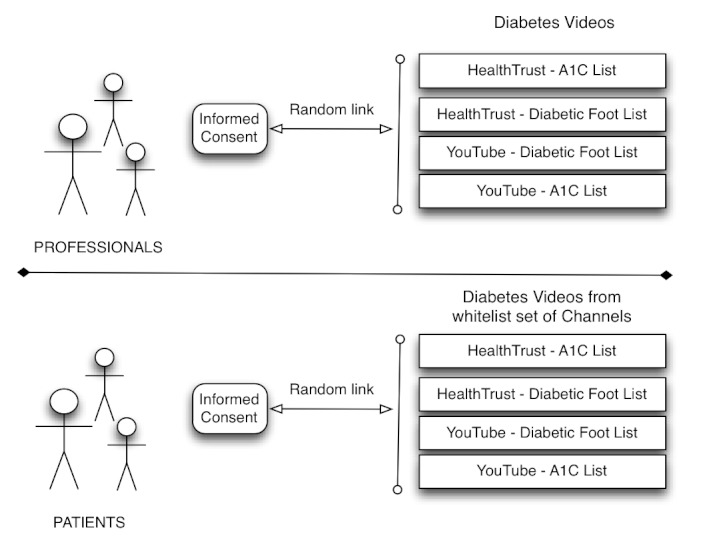
Process of obtaining informed consent from health care professionals and health consumers, and survey allocation.

We evaluated the top 7 video search results for the queries *diabetic foot* and *diabetes A* *1* *c* using both HealthTrust and YouTube search (ordered by relevance). As depicted in Figure 9, after providing informed consent in a Web form the respondents were randomly assigned to a survey with videos to review. Respondents were not informed about the algorithm used to select the videos.

Professional reviewers were assigned to one of four different surveys: two about diabetic foot (one based on YouTube and the other on HealthTrust) and two for hemoglobin A_1__c_ testing. The two lists about diabetic foot for professionals contained 11 videos and the lists about hemoglobin A_1__c_ testing contained 12 videos. Health consumers were assigned to lists for the same queries, but the listed videos were limited to those published by a whitelist of trusted sources. The main reason for using a whitelist was to avoid showing misleading and disturbing videos to consumers (eg, videos featuring amputations). These lists for health consumers contained a total of 17 videos, 8 about diabetic foot and 9 about hemoglobin A_1__c_ testing.

Professionals and health consumers were asked to respond to the following questions about the videos with a Likert scale (eg, from strongly agree to strongly disagree): “Would you recommend this video to a patient with diabetes and questions about diabetic foot?” (question for professionals for a video from the list about diabetic foot); and “Do you like this video about diabetic foot?” (question for health consumers for a video from the list about diabetic foot).

#### Data Analysis

We evaluated the retrieved results using the metrics precision at K [40] and DCG [41]. However, we did not calculate either of these metrics for the health consumers, as they had a prefiltered dataset.

In addition, we used the Pearson correlation [42] to study the correlation between the HealthTrust scores and the average ratings. Pearson correlation is commonly used to study linear dependence between two variables, and the correlation coefficient ranges from −1 to 1. The Pearson correlation was calculated using the psych package of statistical framework R [39].

## Results

### Study of Diabetes Channels

The first study was designed to evaluate the feasibility of using the HealthTrust metric to filter YouTube diabetes channels (aka users). We studied precision at K (K = 5, K = 10, and K = 20) in the top 20 diabetes channels retrieved by the YouTube- and HealthTrust-based searches.

We proposed two possible scenarios for considering a channel relevant: (1) both reviewers recommended the channel and, (2) at least one reviewer recommended the channel. Table 1 shows that the search based on HealthTrust scores performed better than YouTube search in all cases and was only equally good for precision at K = 5 and for channels recommended by both reviewers. The DCG evaluation (Table 2) also resulted in better scores for HealthTrust than for YouTube searches.

**Table 1:** Evaluation of the top 20 diabetes channels by precision at K metric

Recommended by/precision at K	Both reviewers				At least one reviewer			
	YouTube		HealthTrust		YouTube		HealthTrust	
	n	%	n	%	n	%	n	%
K = 5	4	80%	4	80%	4	80%	5	100%
K = 10	6	60%	7	70%	7	70%	9	90%
K = 20	10	50%	13	65%	12	60%	17	85%

**Table 2:** Evaluation of the top 20 diabetes channels by discounted cumulative gain (DCG) metric

Recommended by/DCGi^a^	Both reviewers		At least one reviewer	
	YouTube	HealthTrust	YouTube	HealthTrust
i = 5	2.9	3.1	2.9	3.6
i = 10	3.6	4.1	4	4.9
i = 20	4.6	5.7	5.3	7

^a^ i = number of retrieved videos.

To consider and analyze the capacity of the algorithms to filter out bad content or spam, we considered a channel to be misleading if none of the reviewers recommended it. HealthTrust’s approach performed quite well, filtering out bad channels. For K = 20, HealthTrust’s list had only 3 bad channels (15%) versus 8 (40%) on the YouTube list. In the top 10 channels, HealthTrust had only 1 bad channel (10%) versus 3 (30%) for YouTube. Within the top 5 channels, all HealthTrust’s channels were recommended by at least one reviewer, one more than YouTube.

In the YouTube top 20, some channels featured commercials of diabetes products (eg, testing supplies), several were about a famous diabetic singer (Jonas), and one channel was in Dutch (even though we restricted the search to English in the API). The YouTube list also contained some channels with the word diabetes in its name, but most of the videos were not related to diabetes.

The HealthTrust list did not contain any channels with advertising, but it did have some channels from e-patients with very heterogeneous quality. Surprisingly, some diabetes channels run by public authorities, such as the Juvenile Diabetes Research Foundation, were not highly ranked in HealthTrust. The most logical explanation for this is that some relevant channels do not encourage social interactions (eg, friendships or subscriptions), and this less-connected nature may decrease their rankings.

### Study of Diabetes Videos

In the second study, we explored how the HealthTrust metric can be used to retrieve diabetes videos and also the correlation between HealthTrust and the video’s ratings.

#### HealthTrust Search Evaluation

We calculated precision at K for the list created for professionals to evaluate the performance of the search algorithm. However, we did not study precision at K for consumers, since the dataset was prefiltered.

Precision at K (K = 3, K = 7) for the professionals’ lists was considered as a video rating equal to or greater than 3.5 (values range from 1 to 5). As shown in Table 3, precision was better in HealthTrust for both the diabetes A_1c_ and diabetic foot lists. In the case of diabetic foot, the YouTube list precision was below 50% for both the top 7 and the top 3. The HealthTrust-based search also performed better based on the DCG metric (Table 4).

**Table 3:** Precision at K for videos evaluated by professionals retrieved by HealthTrust and YouTube

Precision at K	Hemoglobin A_1c_				Diabetic foot			
	YouTube		HealthTrust		YouTube		HealthTrust	
	n	%	n	%	n	%	n	%
K = 3	2	66%	3	100%	1	33%	2	66%
K = 7	4	57%	5	70%	3	43%	4	57%

**Table 4:** Discounted cumulative gain (DCG) for videos evaluated by professionals retrieved by HealthTrust and YouTube

DCGi^a^	Hemoglobin A_1__c_		Diabetic foot	
	YouTube	HealthTrust	YouTube	HealthTrust
i = 3	1.6	2.6	1	2
i = 7	2,6	3.4	1.9	2.9

^a^ i = number of retrieved videos.

#### HealthTrust and Rating Correlation

The study of the correlation between HealthTrust score and average rating was used to determine whether our trustworthiness score had a positive impact on the ratings.

For both professionals and consumers, we created two subsets with the videos of each topic (hemoglobin A_1__c_ testing and diabetic foot). We normalized the average ratings of the videos between 0 and 1 for the subset with the videos about hemoglobin A_1__c_ testing and the subset about diabetic foot. Similarly, we normalized the HealthTrust scores within each subset. As shown in Table 5, we compared average ratings and HealthTrust scores for each subset using the Pearson correlation (alpha = .05).

**Table 5:** Pearson correlation between ratings and HealthTrust

	Hemoglobin A_1__c_		Diabetic foot	
	Pearson *r*	*P* value	Pearson *r*	*P* value
Professionals	*r*_10_ = .646	.02	*r*_9_ = .275	.41
Health consumers	*r*_7_ = .649	.06	*r*_6_ = –.019	.96

In the case of the hemoglobin A_1__c_ videos, we found a positive and statistically significant correlation for the professionals’ subset (Pearson *r*_10_ = .646, *P* = .02). This correlation was weaker with the health consumers but still close to significance levels (*r*_7_ = .649, *P* = .06). In the case of the diabetic foot videos, we did not find a statistically significant result in any of the subsets (professionals and consumers).

## Discussion

### HealthTrust Metric Performance

**Figure 10:**
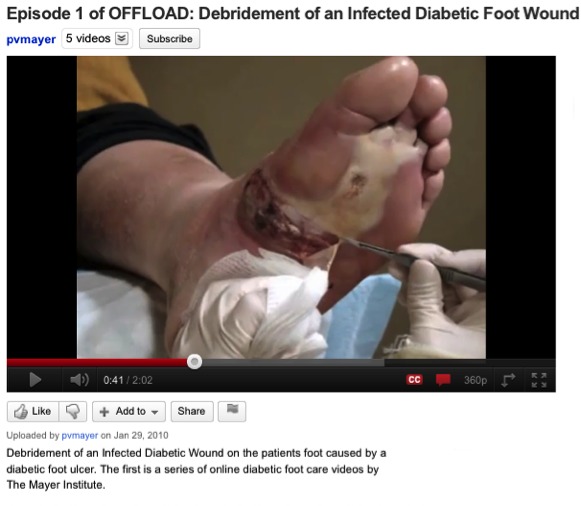
Highly ranked YouTube video about diabetic foot featuring an infected wound.

Our results suggest that social network analysis may be used to gather information about the quality of health information. The retrieval of diabetes videos and channels based on the HealthTrust metric performed reasonably well, compared with the YouTube search. In nearly all cases, the precision of the lists retrieved using HealthTrust was better than those retrieved using YouTube. Precision is very important, since in the health-irrelevant content can be potentially very negative (see Figure 10). It is quite significant that the performance of HealthTrust was equal to or better than that of the YouTube search, considering that YouTube has access to all the metadata about videos and users, while HealthTrust has limited access via its API; for example, some channels restrict access to information about their links (eg, subscriptions).

It is difficult to identify the exact differences between the YouTube and HealthTrust searches, since YouTube has not published its search algorithm, despite having published its recommended algorithm [43]. However, we expect YouTube’s search engine to be based on link analysis, as are most search engines. The main difference between traditional Web search engines and our approach is that we strengthened the tightly knit community effect [19], as with diabetes; traditionally, Web search engines try to reduce the influence of those communities to raise general public satisfaction. Consequently, funny or controversial videos are more popular among the general YouTube community and are therefore more highly rated. These videos lose prominence using HealthTrust. In fact, the search based on the HealthTrust metric performed better than YouTube in filtering out misleading videos (eg, herbal cures or advertisements). The HealthTrust algorithm estimates health-related trust and not general trust on YouTube.

### HealthTrust Weaknesses

Some good videos from trusted sources, such as public health authorities, gained relatively low HealthTrust values. The best explanation for the algorithm’s behavior is that creators of those videos had fewer connections in the diabetes community. Some of these sources belonged to a more generic health community (eg, CDCStreamingHealth’s Channel) and therefore had weaker ties with the diabetes community. Also, some trusted sources do not create links with other users (eg, through friendships, subscriptions, or comments). This lack of connectivity leads to lower scores in HealthTrust. As part of our future work, we will design an enhanced version of HealthTrust that calculates trustworthiness values within several health communities.

**Figure 11:**
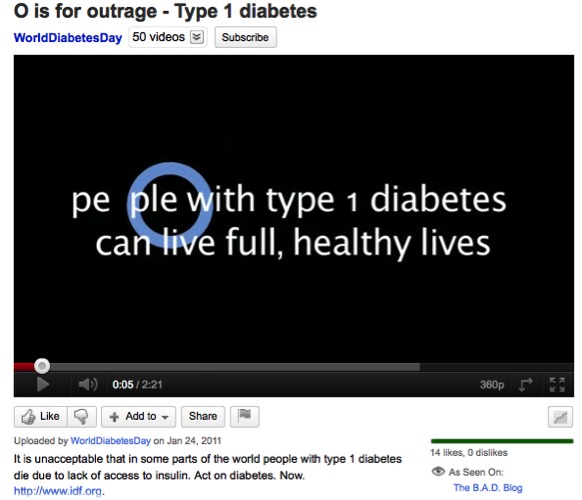
Screenshot from the video *O is for outrage – Type 1 diabetes*.

Many factors influence the perceived quality of a video beyond trustworthiness and authoritativeness. Therefore, it is not surprising that we did not find statistically significant correlations in all cases. Personal taste and preferences play a major role. For example, the video *O is for outrage – Type 1 diabetes* (Figure 11) was given a higher rating by health consumers (average of 4.2) than by professionals (average of 2.75). *O is for outrage* is a video appealing to emotional aspects to raise awareness; it is very engaging to the online diabetes community. However, this particular video is less informative, which may explain why professionals rated it lower. Consequently, a generic quality indicator such as HealthTrust cannot always satisfy everybody.

There were videos from certain channels with quite different average ratings but the same HealthTrust scores. In such cases, the videos had no links (favorites) but inherited the HealthTrust scores from their channels. An example of this problem is shown in the following two videos from the diabetic foot list for consumers: (1) *Baseball great Ron Santo & Diabetes--INCREDIBLE Story*, and (2) *Miami Ink* *’* *s Darren Brass: Tattoos and Diabetes*. Both videos have the same HealthTrust score, as both are from the same diabetes channel, dLifedotcom. However, the *Miami Ink* video was less appealing to health consumers. In this case, link analysis was not enough to distinguish the quality between the two videos. The only way to solve this problem is to analyze more data about the video (eg, semantic analysis or ratings).

### Limitations

In both experiments, some videos or channels were deleted while we were conducting the experiment. In the case of the channel study, two were removed, and in the second study some videos gained lower ratings because they were made private by their authors. It is unlikely that this problem biased our study, since it affected a small sample and it affected all the algorithms equally.

To be able to generalize our findings, we will have to perform large-scale studies with more queries, reviewers, and videos. Our survey-based evaluation approach is merely an approximation of the real context of health consumers’ search for information. Survey-based evaluation of online videos is very time consuming, as most videos last several minutes. It was necessary to watch around 30 minutes of videos to complete our surveys. Hence, to generalize our findings we are implementing a video portal to capture more data for evaluation within the real user context. The video portal will also need to address the continuous changes in the structure of online communities (eg, reputation changes over time). A possible solution for the dynamic nature of online communities may be periodic calculation of HealthTrust.

Moreover, it remains to be seen whether our approach will work in health domains where there is a large community of users promoting misleading information. For example, there are communities promoting anorexia as a lifestyle [15] or against vaccination [14]. Pro-anorexia users will tend to link and endorse misleading information; thus, if HealthTrust is to be used to retrieve trustworthy content about anorexia it must be able to avoid pro-anorexia subcommunities.

Our current study is limited to online health videos; therefore, we will need to replicate our study with other types of social media in order to generalize our findings. We believe that the metric HealthTrust can be applied to any type of linked health community where users are interconnected via follows, friendships, and favorite content. However, experiments will need to be performed to evaluate the algorithm, since each type of community may have a different structure and dynamics.

Our study is limited to automatic approaches for extracting trust-based metrics and the feasibility of using these metrics to retrieve health videos. More research will be needed to test how to combine HealthTrust with manual selection of social media by human experts. HealthTrust can be very useful to automatically identify the most trusted sources within the diabetes community. However, some trustworthy providers have very good content but have not gained enough trust within the online community.

### Conclusions

Every day, millions of health consumers search for health information on social platforms such as YouTube, and retrieving accurate information from trusted sources can often be difficult. There is an unsatisfied need for new information retrieval tools to help health consumers find trustworthy and relevant health information within social media.

In this paper we present a new metric, called HealthTrust, to infer information about the trustworthiness of social media content within a health community. We tested the feasibility of using HealthTrust for retrieving videos from the diabetes community on YouTube. Based on our evaluation with health consumers and professionals, the search of diabetes content based on the HealthTrust metric performed better than YouTube in nearly all the tested cases. However, a larger study is needed to validate our results in a health portal in order to test the metric in a live setting.

Despite the limitations of our study, we conclude that, to apply social network analysis to retrieving health information, social media may be used to develop tools that will ultimately help find relevant and trustworthy information. Social network analysis could also be used to reinforce other approaches to health information retrieval such as quality labels and manual review of content.

## Abbreviations

API: application programming interface
DCG: discounted cumulative gain
CDC: Centers for Disease Control and Prevention
HITS: hyperlink-induced topic search
